# Electroconvulsive Therapy in Super Refractory Status Epilepticus: Case Series with a Defined Protocol

**DOI:** 10.3390/ijerph17114023

**Published:** 2020-06-05

**Authors:** Beatriz García-López, Ana Isabel Gómez-Menéndez, Fernando Vázquez-Sánchez, Eva Pérez-Cabo, Francisco Isidro-Mesas, Arturo Zabalegui-Pérez, Ignacio Muñoz-Siscart, María Carmen Lloria-Gil, Raúl Soto-Cámara, Jerónimo J. González-Bernal, Josefa González-Santos, José M. Aguilar-Parra, Rubén Trigueros, Remedios López-Liria, Troels Wesenberg Kjær

**Affiliations:** 1 Neurophysiology Department, Burgos University Hospital, 09006 Burgos, Spain; bgarcialo@saludcastillayleon.es (B.G.-L.); agomm@saludcastillayleon.es (A.I.G.-M.); fisidrom@saludcastillayleon.es (F.I.-M.); mclloriag@saludcastillayleon.es (M.C.L.-G.); 2 Neurology Department, Burgos University Hospital, 09006 Burgos, Spain; fvazsan@saludcastillayleon.es; 3 Intensive Care Unit, Burgos University Hospital, 09006 Burgos, Spain; eperezcab@saludcastillayleon.es (E.P.-C.); azabalegui@saludcastillayleon.es (A.Z.-P.); 4 Psychiatry Department, Burgos University Hospital, 09006 Burgos, Spain; imunozs@saludcastillayleon.es; 5 Department of Health Sciences, University of Burgos, 09001 Burgos, Spain; 6 Department of Psychology, Health Research Centre, University of Almeria, 04120 Almeria, Spain; jmaguilar@ual.es (J.M.A.-P.); rtr088@ual.es (R.T.); 7 Department of Nursing, Physiotherapy and Medicine, Health Research Centre, University of Almería, 04120 Almeria, Spain; rll040@ual.es; 8 SjællandsUniversitets Hospital, 4000 Roskilde, Denmark; twk@regionsjaelland.dk

**Keywords:** electroconvulsive therapy, ECT, super refractory status epilepticus, antiepileptic treatment

## Abstract

Super-refractory status epilepticus (SRSE) represents a neurological emergency that is characterized by a lack of response to the third line of antiepileptic treatment, including intravenous general anesthetics. It is a medical challenge with high morbidity and mortality. Electroconvulsive therapy (ECT) has been recommended as a nonpharmacologic option of treatment after other alternatives are unsuccessful. Its effect on the cessation of SRSE has been minimally investigated. The objective of this article is to analyze the effect of ECT on SRSE. For this purpose, a multidisciplinary team created a protocol based on clinical guidelines similar to those described previously by Ray et al. (2017). ECT was applied to six patients with SRSE after the failure of antiepileptic treatment and pharmacologic coma.The objective of each ECT session was to elicit a motor seizure for at least 20 s. SRSE was resolved in all patients after several days of treatment, including ECT as a therapy, without relevant adverse effects. Thus, ECT is an effective and feasible option in the treatment of SRSE, and its place in the algorithm in treatment should be studied due to the uncommon adverse effects and the noninvasive character of the therapy.

## 1. Introduction

Classically, status epilepticus (SE) is defined as “a condition characterized by an epileptic seizure that is sufficiently prolonged or repeated at sufficiently brief intervals so as to produce an unvarying and enduring epileptic condition”. A special report by the International League Against Epilepsy (ILAE) proposes the following SE definition that encompasses all types of SE and takes into consideration the current knowledge regarding the pathophysiology and the need to address the clinical treatment decision: “a condition resulting either from a deficiency of the mechanisms responsible for the cessation of the crisis or from the beginning of mechanisms that lead to abnormally prolonged seizures (after time point t_1_). It is a condition that can have long-term consequences (after time point t_2_), including neuronal death, neuronal injury, and alteration of neuronal networks, depending on the type and duration of seizures” [1]. Refractory SE (RSE) refers to SE that persists despite the administration of at least two appropriately selected and dosed parenteral medications, including benzodiazepines [2]. Finally, it is considered as super-refractory SE (SRSE) when the SE persists for at least 24 h after the onset of anesthesia, either without interruption despite appropriate treatment with anesthesia, recurring while on appropriate anesthetic treatment, or after the withdrawal of anesthesia and requiring anesthetic reintroduction [2].

SE represents a neurological emergency that is commonly seen in clinical practice. Its annual incidence is around 35 cases per 100,000, decreasing to 7.2 for RSE and 1.2 for SRSE [3], with unequal distribution by age, being more common in children and older adults [4,5,6]. It is a severe medical condition with significant associated morbidity and high mortality rates of up to 33–38%, depending on the etiology, study population, and type of seizures, being substantially higher for RSE and SRSE [4,7,8]. Despite optimum treatment, up to 10% of the SE cases admitted to a hospital will become SRSE [9], accounting for 4% of seizure-related hospital discharges [10]. Regarding morbidity, prolonged duration of the patient’s hospital stay has been correlated with a poor functional outcome at one-year follow-up [11]. New functional defects have been reported in a one-year follow-up in 30% of the patients with RSE, without a poorer outcome in SRSE cases. The outcome was worse in older patients and in those with progressive or fatal etiologies [12].

As the underlying cause of SE has a great influence on the outcome, an attempt should be made to identify it whenever possible [13,14]. Both SE and RSE occur more frequently in patients with known epilepsy.

An imbalance in the function of the cortical excitatory and inhibitory neurons or a failure of normal inhibitory mechanisms has been hypothesized to be among the possible pathophysiological reasons for SE and its refractoriness. Gamma-aminobutyric acid (GABA) is the most common inhibitory neurotransmitter, preventing neurons from excessive excitation by activation of the GABA receptors. Glutamate is the most common excitatory neurotransmitter, which mediates excess excitation via the *N*-methyl-d-aspartate (NMDA) receptors. When these conditions are maintained over time, they cause neuronal death due to an increased concentration of intracellular Ca^2+^ and reorganization of the brain’s net circuits [15,16].

The primary objectives of treatment against SE are controlling seizures to prevent the initial process of excitotoxicity and their downstream consequences, protecting brain function to the maximum possible extent, and avoiding or treating the complications due to the multisystem dysfunction resulting from ongoing seizures, use of anesthetics, prolonged loss of consciousness, and immobility [17].

Several drugs, such as antiepileptics, benzodiazepines, ketamine, inhaled anesthetics, immunoglobulins (Ig), steroids, or immunomodulators, and new modalities of nonpharmacological therapies, such as ketogenic diet, hypothermia, plasmapheresis, neuromodulatory techniques, or surgery, have been used in the treatment of RSE and SRSE [17,18,19,20,21].

Several neuromodulatory techniques, such as electroconvulsive therapy (ECT), implanted direct vagal nerve stimulation (VNS), transcranial magnetic stimulation (TMS), or deep brain stimulation (DBS), have shown promising results in series and case reports. However, the quality of currently available evidence supporting their use against RSE or SRSE is low [21,22].

ECT involves transcutaneous electrical stimulation of the cerebral cortex in order to induce a seizure-like episode [23,24,25]. It has been widely applied in refractory psychiatric diseases, such as depression or schizophrenia, with medically documented safety and efficacy [23,24,26]. Its benefits on seizure-based disorders have been hypothesized to be associated with an alteration in neurotransmitter levels, elevation in seizure threshold posttreatment, changes in prolactin levels, promotion of neurotrophic factors, and their role on synaptic neuroplasticity and decrease in neural metabolism [27,28].

The objective of this article is to analyze the role of ECT in SRSE, describing a prospective case series and the protocol used.

## 2. Method

### 2.1. Study Design and Participants

This study was a prospective case series, commonly known as anecdotal reports, case study research, or clinical reviews, with six patients at the Burgos University Hospital (Spain).

Informed consent was obtained from a close relative of the patients. We obtained approval from the appropriate Institutional Review Board (Burgos University Hospital IRB1860) to apply ECT as previously described in the literature: as a therapeutic measure in treatment against SRSE, as an adjuvant of antiepileptic drugs (AED) and anesthetic treatment, and after the failure of the first anesthetic or barbituric coma. The rules and recommendations described in the Helsinki Declaration were complied with at all times.

### 2.2. Procedure

After the first case of a child with Febrile Infection-Related Epilepsy Syndrome (FIRES), in which ECT was used in our hospital as the final and desperate treatment after two months of SRSE, published by Mirás-Veiga et al. [29], the need for a protocol for these cases was noticed. A multidisciplinary team was established for this purpose.

After a detailed review of the existing literature, and similar to the protocol described by Ray et al. for possible future studies using ETC on SRSE patients [30], we prepared a working protocol specific for our population and our hospital. The aim was to apply ECT in patients with SRSE, after failure to control the disorder by at least two antiepileptic treatments (at least three in our hospital), including benzodiazepines and general intravenous anesthesia, at adequate dosage [30].

### 2.3. Intervention: ECT Protocol

Given the fact of being in SRSE condition and having shown the effect of pharmacologic coma (anesthetic or barbituric), antiepileptic treatment was maintained, although optimized at a minimally effective dosage, to try to elicit a motor seizure from each ECT session. In addition, the ECT session was adapted in time, so that the AED had not been administrated yet. Patients were under anesthetic treatment during these days, which was stopped before the daily ECT session (propofol for 30–60 min and midazolam for at least 3–4 h). Specifically, fentanyl was used as an analgesic during the ECT session. The patients were kept on solid fast and liquid fast for 8 and 4 h, respectively, or were fed by nasogastric tube with adequate oxygenation by maintaining optimum ventilation. The electrodes were placed on bilateral frontotemporal regions that were previously cleaned with alcohol and conductive gel. The ischemic cuff was applied before the administration of muscle relaxants. A teeth protector was used. The objective of each ECT session was to elicit a motor seizure of at least 20 s. Up to three stimuli were provided per ECT session to achieve this objective. The intensity of the initial stimulus was 500 mC. The intensity was gradually increased up to a maximum of 1000 mC (maximum available) if supplementary stimuli were required. The frequency of stimulation was 60 Hz, and the duration of the stimulus was 5 s. If an ECT session went without generating a motor seizure response, oral 300 mg caffeine was administrated, which was increased to 500 mg for the next session, if the former did not achieve motor response either. The acoustic stimulus was performed just before electrical stimulation to facilitate the recruitment of brain activity in some cases. ECT was applied on a daily basis with a few exceptions.

The cerebral activity was monitored by video-electroencephalography (V-EEG), with the electrocautery filter to protect the amplifier. Long-term V-EEG was performed on a daily basis to monitor brain activity from 45 min before ECT to 1–3 h after. On EEG recordings, rhythmic discharge or spike-and-wave pattern with definite evolution in frequency, location, or morphology lasting at least 10 s was considered ictal activity. No convulsive SE (NCSE) was defined according to the modified Salzburg Consensus Criteria [31,32,33]. The pre-ECT EEG defined the presence of SRSE in the appropriate clinical context on the first day and assessed its evolution over the following days. EEG recording during and after ECT was done continuously for up to 3 h after each ECT session, reintroducing the anesthetics at minimum dosage when the epileptic discharges reappeared. Each case was individually discussed, and the ECT was stopped when the SRSE was resolved. In the clinical evolution of patients, SRSE was considered to be resolved when the continuous epileptiform activity (>90%) disappeared, and the Salzburg criteria for NCSE were not fulfilled [31,32,33,34].

## 3. Results

**Figure 1:**
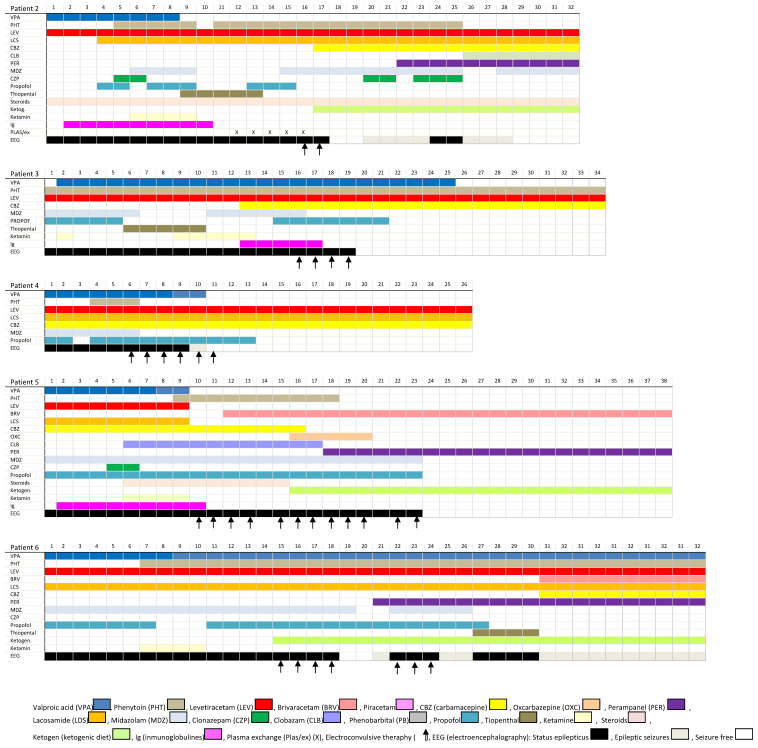
Treatments of each patient from accepting in ICU or 15 days before the first ECT after the last ECT.

**Figure 2:**
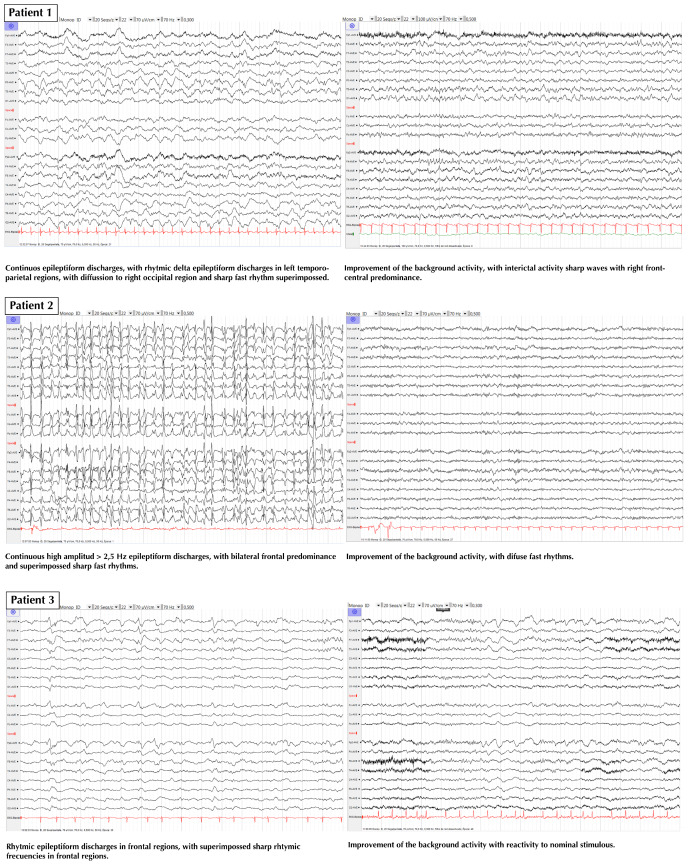

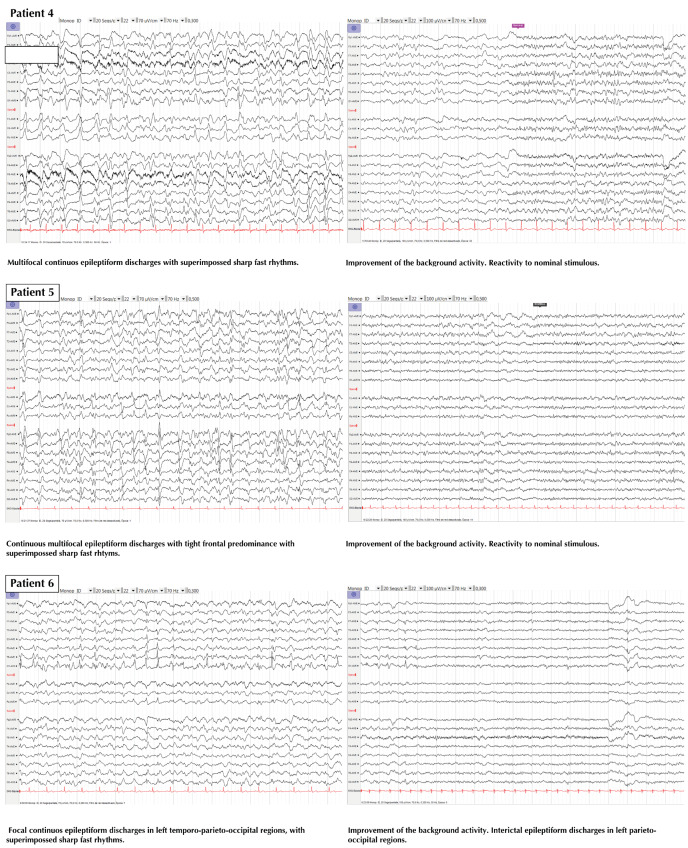
EEGs of all patients. Left image: EEG in the ICU just before the first ECT session; Right image: EEG the day after the last ECT session.

We report all the cases that were treated with ECT at our center, attempting to avoid the bias of anecdotal reports where only successful cases are reported. The cases are briefly summarized, and a description of their treatment is presented in Figure 1. Two representative EEGs of each patient (just before the first ECT session and the day after the last session) are presented in Figure 2 and in a bigger format in supplementary material (Figure S1).

Patient 1

A 4-year-old male child with no relevant medical history. Normal development until hospitalization. He suffered from amygdalitis, with a fever of up to 40.5 °C. Six days later, he developed an ataxic gait with motor epileptic aphasia with tonic-clonic evolution leading to SRSE. He was hospitalized in the intensive care unit (ICU). EEG showed continuous epileptic discharges, with delta rhythmic epileptiformdischarges ofspatio-temporal evolution and ictal clinical phenomena. Magnetic resonance imaging (MRI) of the brain was normal. The diagnosis was FIRES. He was the first patient of our series to be treated with ECT. This therapy was initialized at day60 of the onset of SRSE. SRSE was resolved after seven sessions of ECT. He was discharged from the hospital with severe cognitive-motor sequelae and severe epileptic encephalopathy.

Although this patient was treated prior to standardizing the protocol, we considered this case to be relevant to the effect of ECT that we have attempted to study here, due to which we have included this patient’s clinical information and treatments as described in Figure 1, but this case was excluded from the analysis of protocol data.

Patient 2

A 32-year-old woman with no relevant medical history. She was hospitalized due to intense otalgia associated with fever. During hospitalization, she developed opsoclonus-myoclonus, and then showed impaired consciousness. Cranial nerve palsy involving left IX to XII nerves was observed. She initially developed focal motor seizures of the left half of the body, which rapidly evolved to multifocal SE. She required hospitalization in the ICU. EEG showed continuous high amplitude >2.5 Hz epileptiform discharges and focal motor seizures from the right inferior frontal region at onset, with typical spatio-temporal evolution. Brain MRI showed mild hypersignals of bilateral putamen, caudate nucleolus, punctiform multiple lesions of the left hemisphere, and enhancement of the left cerebellar tonsil after gadolinium injection. All these conditions were resolved in control MRI. Etiologic large research was negative. As she was unresponsive to medical treatment, ECT was applied, which resolved the status after two sessions. She was diagnosed with NORSE (new onset refractory status epilepticus), associated with clinical brainstem involvement. The clinical situation improved with treatment involving immunotherapy. Two months after discharge, she continued to suffer from auditive focal seizures without conscious impairment every ten days aproximately, and showed moderate memory impairment, as assessed by a detailed neuropsychological evaluation.

Patient 3

A 77-year-old woman with atrial fibrillation. She was treated with warfarin. She had no other relevant medical history. She was hospitalized after sudden enchained tonic-clonic seizures, with no recovery of consciousness between seizures. She was directly intubated and hospitalized in the ICU. EEG showed continuous delta rhythmic epileptiform discharges in the frontal regions, with superimposed sharp rhythmic epileptiform discharges. Brain MRI was normal. Her SRSE, which was unresponsive to all pharmacological treatment, was resolved after four sessions of ECT. She was diagnosed with NORSE. She was discharged and is living a normal life without sequelae.

Patient 4

A 75-year-old man with a medical history of colorectal cancer in remission and left temporal lobe epilepsy. He was treated with 1500 mg levetiracetam twice a day. He also has one seizure every five years. He was hospitalized in the psychiatry department due to an autolytic attempt. He was found unconscious in his hospital room. EEG showed continuous focal epileptiform discharges >2.5 Hz with generalized frontocentral predominance, with the sharp fast rhythm superimposed. Brain MRI showed isolated white matter lesions compatible with leukopathy. As he was unresponsive to the initial pharmacological treatment, he was hospitalized in the ICU. Six sessions of ECT helped resolve SRSE, with a slow improvement in the mental state based on bedside general neurologic evaluation. He was diagnosed with nonconvulsive focal SRSE secondary to left temporal lobe epilepsy. He died a few weeks later in another hospital due to pneumonia secondary to broncho-aspiration, during which he suffered only one epileptic seizure after discharge.

Patient 5

An 83-year-old woman with a medical history of diabetes with good control under oral treatment and no other complaints. She presented with a sudden behavioral change. EEG showed continuous multifocal epileptiform discharges with right frontal predominance and superimposed sharp, fast rhythms. She was hospitalized in the ICU. MRI showed a recent brain infarct in watershed areas of the anterior and middle-left cerebral arteries. Her SRSE, which was unresponsive to medical treatment, was resolved after 12 ECT sessions. Her mental state improved slowly from bedside neurologic general evaluation, right after exiting from the ICU. The diagnosis was nonconvulsive SE secondary to the left hemispheric brain infarct. She died 15 days after discharge from our hospital due to comorbidity associated with prolonged hospitalization in a convalescence center.

Patient 6

A 51-year-old man with a medical history of severe cranial trauma with traumatic epilepsy due to a left occipital lesion that was secondary to the hemorrhagic lesion. He also had mixed anxiety-depressive disorder as a pathologic antecedent. He was treated with unknown AED without medical compliance and hospitalized after one episode of tonic-clonic seizure, followed by aphasia lasting for several hours. EEG showed focal continuous epileptiform discharges in left temporo-parieto-occipital epileptiform discharges, with typical ictal spatio-temporal evolution. As he was unresponsive to initial medical treatment, he was admitted to the ICU. SRSE was resolved after seven sessions of ECT. MRI of the brain showed a residual left occipital lesion that was unchanged compared to the previous neuro-image. He was discharged three months later with cognitive impairment, mainly aphasia. Follow-up was not possible as the patient neither reported for consultation nor answered phone calls after discharge.

Patients with RSE were admitted to the ICU after a variable timing of conventional hospitalization. From the time of admission to ICU, ECT was applied within the first 15 days (mean time of 12.8 days). We have excluded Patient1 from this analysis as he was the first patient to which ECT was applied as a desperate treatment measure, and he was not included in the prospective protocol described, which was used for the rest of the patients. As evident in Figure 1, other treatments were administrated before and during the ECT sessions. In Patient3, propofol was reintroduced around the same time as ECT. In Patient5, a ketogenic diet and clobazam were initiated after ECT.

The number of ECT sessions varied in the series, from 2 to 12 per patient, with a mean of 6.3 sessions, and was administrated on a daily basis with few exceptions. Only Patient4 showed complete cessation of SRSE and seizures after six sessions of ECT, while no other treatment escalations were made. In Patients 1, 2, and 6, SRSE was resolved (Patients 1 and 6 after seven sessions; Patients 2 and 3 after two and three sessions, respectively), although epileptic seizures continued (the continuous epileptiform activity (>90%) disappeared, and NCSE criteria of Salzburg were not fulfilled [33,34]).

None of the patients had a motor seizure response on the first day of ECT sessions. When a motor seizure was achieved, it was mostly in the first stimulus. Many ECT sessions did not cause a motor seizure; nevertheless, the control of SRSE was obtained in all patients. Regarding the effect of ECT on the EEG activity, besides the provocation or not of a motor seizure, a global attenuation of frequencies was observed for several seconds just immediately after the ECT. During this attenuation, there was a lack of the epileptic activity registered before the ECT. After this brief time (seconds), the epileptic activity reappeared, and anesthetic treatment was reintroduced until the next day. Regarding epileptic activity after discharge from the ICU, none of the patients had a focal motor seizure, nor a generalized tonic-clonic seizure in their clinical evolution. Only focal nonconvulsive seizures were observed, indicating a significant improvement compared to SRSE, without relevant adverse effects.

## 4. Discussion

Administering ECT has been recommended to cases of SRSE as a viable treatment option when all the others are unsuccessful. However, there are only a small number of studies describing its application for SRSE [23,35]. This low use in the management of SRSE may be related to a lack of data to support it, limited availability in the ICU, and inexperience of the most neuro-intensivists [19,23]. A systematic review has reported a success rate of approximately 80% in the control of RSE [25]. Aspects such as the placement of electrodes or parameters of electrical stimulation modulate both efficacy and side effects of the ECT [25].

Similar to us, some authors have expressed their concern about a better outcome if the ECT had been applied earlier [25,29,30,36,37], and have suggested that ECT should be applied early during the evolution of SE [38]. We tried to apply ECT as soon as possible after the SRSE was defined. As pointed out by Ray et al. [30], the earlier the intervention, the better would be the result, as there would be less excitotoxic damage and lower downregulation of the inhibitory system of the brain [30], as well as less risk for systemic infection [15]. In fact, once the protocol was available, all the patients (except Patient1) were treated with ECT within an average of 13 days (range 7–16 days) after admission to the ICU.

Another important finding was that ECT showed a positive response regarding the control of SRSE, even in the cases in which it did not achieve a motor seizure response. In fact, some patients did not have an epileptic motor seizure during any ECT session, while the duration of seizures was very brief in others. However, we did observe a global attenuation of frequencies, for a few seconds right after ECT, in all the patients without ECT motor seizure. We hypothesize that the neuromodulatory effect of ECT and at least some of the effects attributed to the therapy, such as the re-externalization of GABA receptors and others, may be independent of the presence of motor seizure induced by ECT. Patient2 had withdrawal seizures and SRSE as recurrence, seven days after ECT. This could possibly be related to insufficient ECT sessions, as she had received the least number of ECT sessions, only two, until an initial complete resolution of SRSE. Previous literature has recommended a variable number of ECT sessions, with around 6 to 12 sessions, depending on the response [30], due to which we are not aware of a minimum of ECT sessions to achieve a more prolonged response to therapy. We think that idiopathic cases with early ECT treatment have a better functional outcome, as only Patients 2 and 3 (idiopathic and early-treated) showed a favorable functional outcome, although more cases should be carefully studied.

Only mild adverse effects were noted, with transient amnesia. However, we must also take into account that the amnesia could be caused by anesthetic drugs, which were taken by all the patients during their stay in the ICU, and also due to SRSE sequela.

### Limitations

The main limitations of our study are its small sample size and the lack of control group, due to which the efficacy of ECT cannot be confirmed from this series. The efficacy of ECT against SRSE should be demonstrated in a crossover study with a larger sample of patients, preferably with a control group. We must consider the inherent severity of SRSE, with its poor outcome and high probability of mortality.

We must note that all the patients that had received AED and anesthetics before and during the days of ECT were allowed for escalation of other therapies (such as new anesthetic infusions, ketogenic diet, or clobazam). Therefore, all these therapies could have individually contributed to resolving SRSE, and the role of each therapy needs to be studied in more detail. Nevertheless, we must remember that ECT in our series was applied after the failure of intensive AED treatment and the occurrence of anesthetic or barbituric coma, which indicates that ECT may actually have resolved SRSE in the short term. Nevertheless, epileptic seizures were observed in half of the patients during its clinical evolution.

## 5. Conclusions

Treatment with ECT has been included as a nonpharmacologic alternative after conventional therapy. We agree with the previous investigators who suggest that ECT is a reasonable and feasible option in the treatment of SRSE, due to the observed time relationship of SRSE resolution following the application of ECT. This fact, along with the rare adverse effects of this noninvasive therapy that is widely used in other pathologies, also makes us reconsider its place in the treatment algorithm of SRSE. We should consider the limitations in assessing Level-I evidence, especially due to the rarity of SRSE cases, which reduces the possibility of performing a case–control study. However, it is our responsibility to collaborate and share methods to achieve this evidence. Therefore, larger, prospective, and multicentric case–control studies are needed to understand the role of ECT in the treatment of SRSE.

## Supplementary Materials

The following are available online at https://www.mdpi.com/1660-4601/17/11/4023/s1, Figure S1: EEG of all patients, the day of the first ECT in the ICU (before the session) and the day after the last ECT session.
