# Supplementary material for: Electroconvulsive Therapy in Super Refractory Status Epilepticus: Case Series with a Defined Protocol
Source: Int J Environ Res Public Health. 2020 Jun 5;17(11):4023. doi: 10.3390/ijerph17114023 (PMC7312395; doi:10.3390/ijerph17114023)
Supplement: Supplementary file 1 [file ijerph-17-04023-s001.pdf]

Patient 1

Upper image: EEG the day of the first ECT, in the ICU (before de session); lower image: EEG the day after the last ECT session.

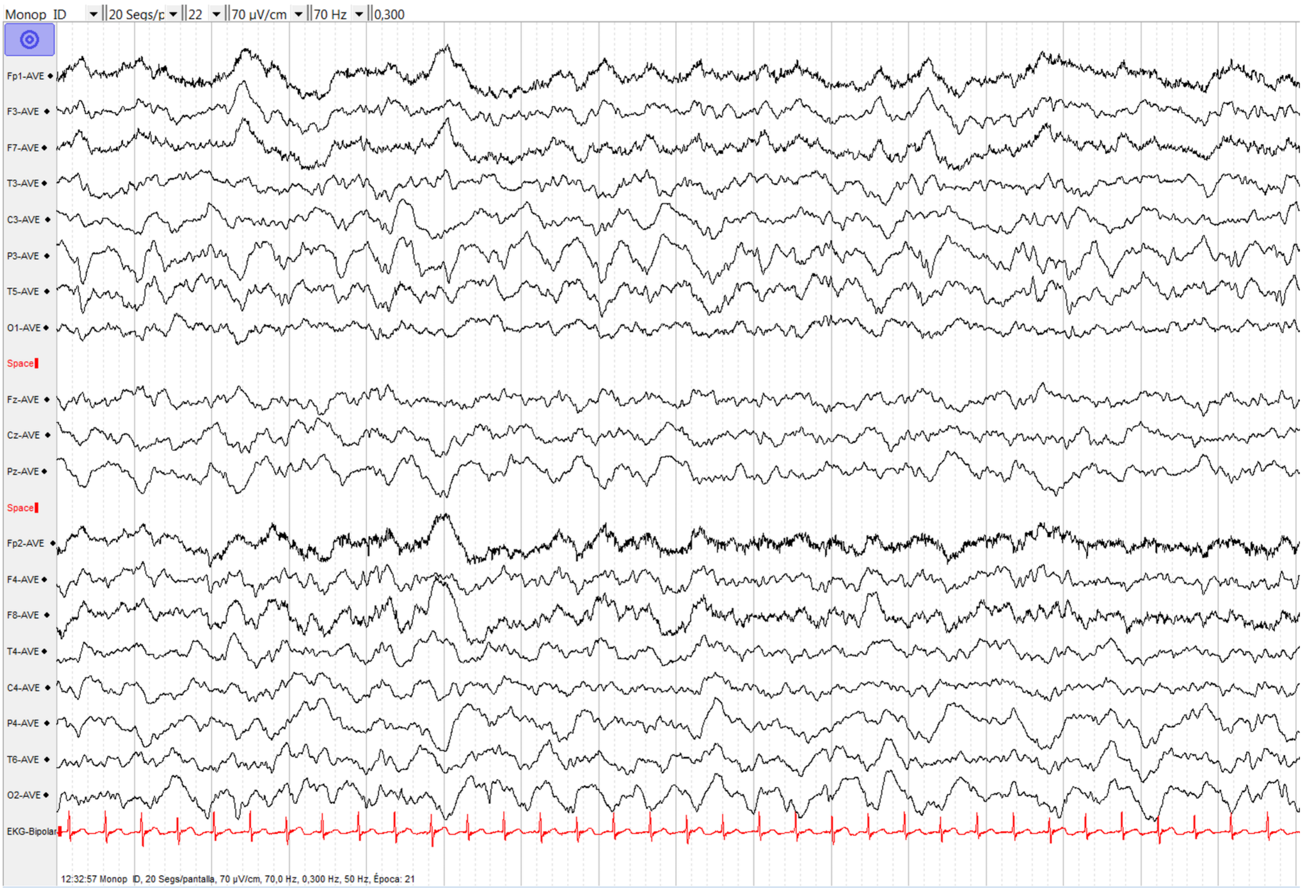

Continuos epileptiform discharges, with rhytmic delta epileptiform discharges in left temporo-parietal regions, with diffussion to right occipital region and sharp fast rhythm superimpossed.

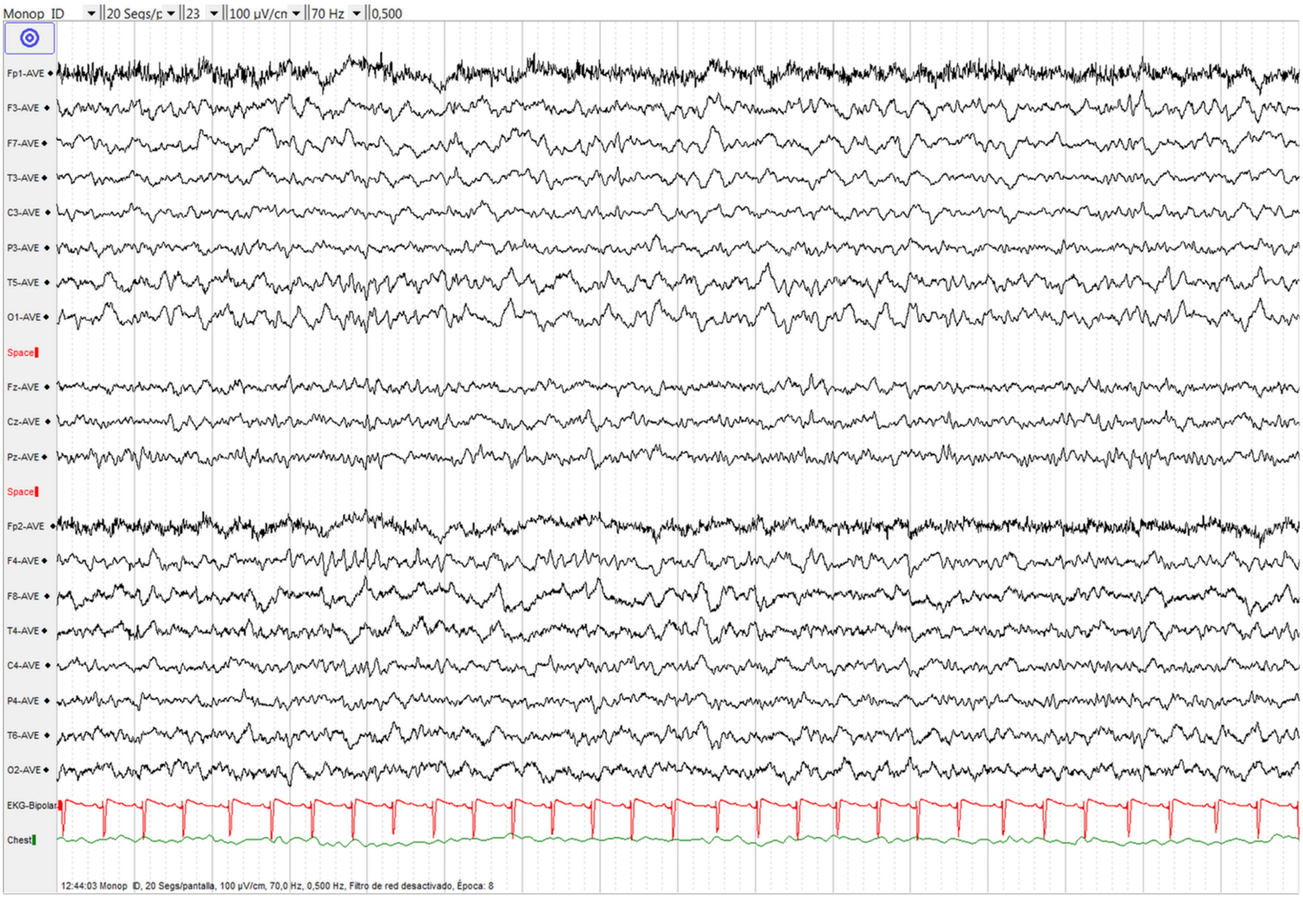

Improvement of the background activity, with interictal activity sharp waves with right front-central predominance.

Patient 2

Upper image: EEG the day of the first ECT, in the ICU (before de session); lower image: EEG the day after the last ECT session.

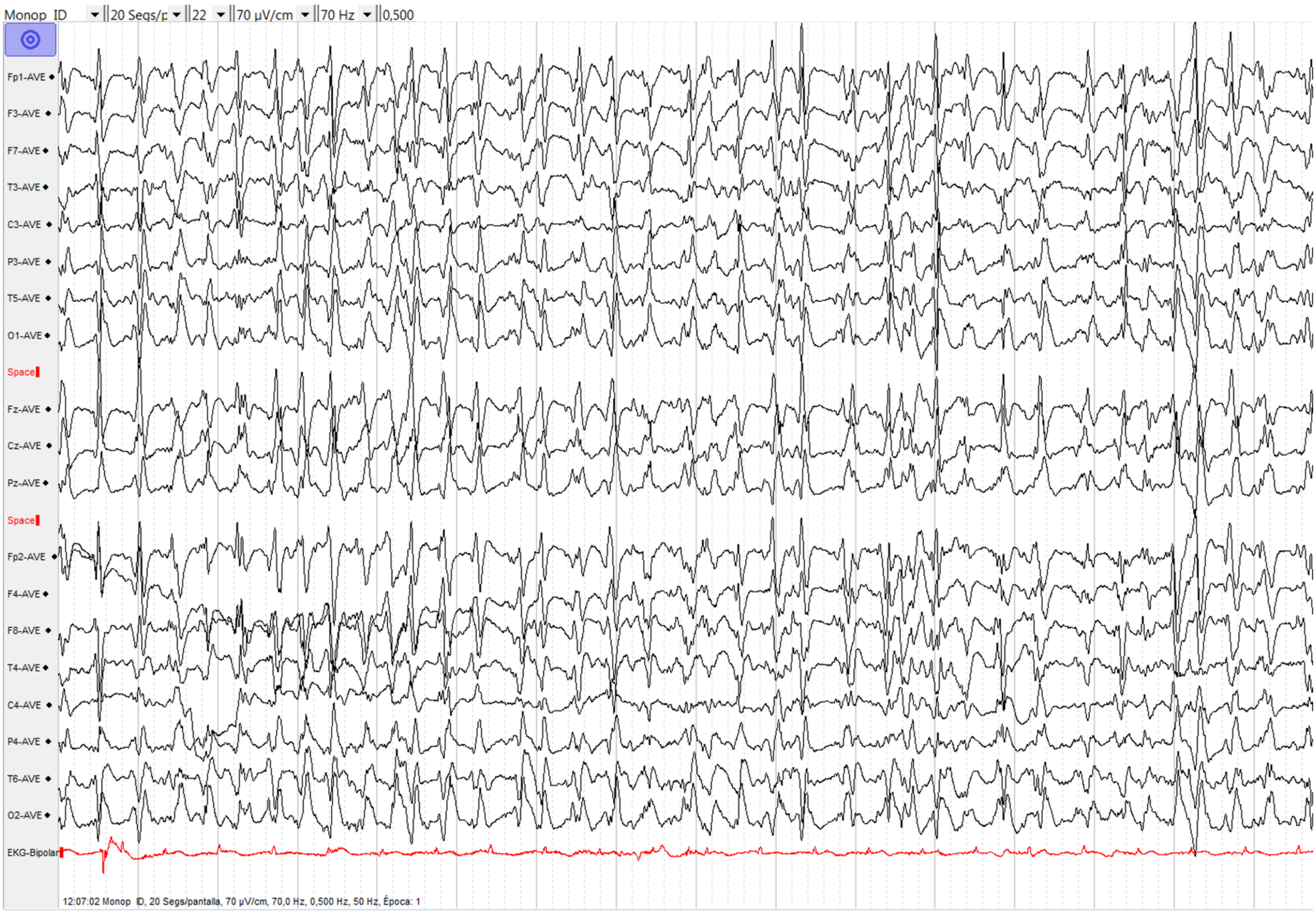

Continuous high amplitud > 2,5 Hz epileptiform discharges, with bilateral frontal predominance and superimpossed sharp fast rhythms.

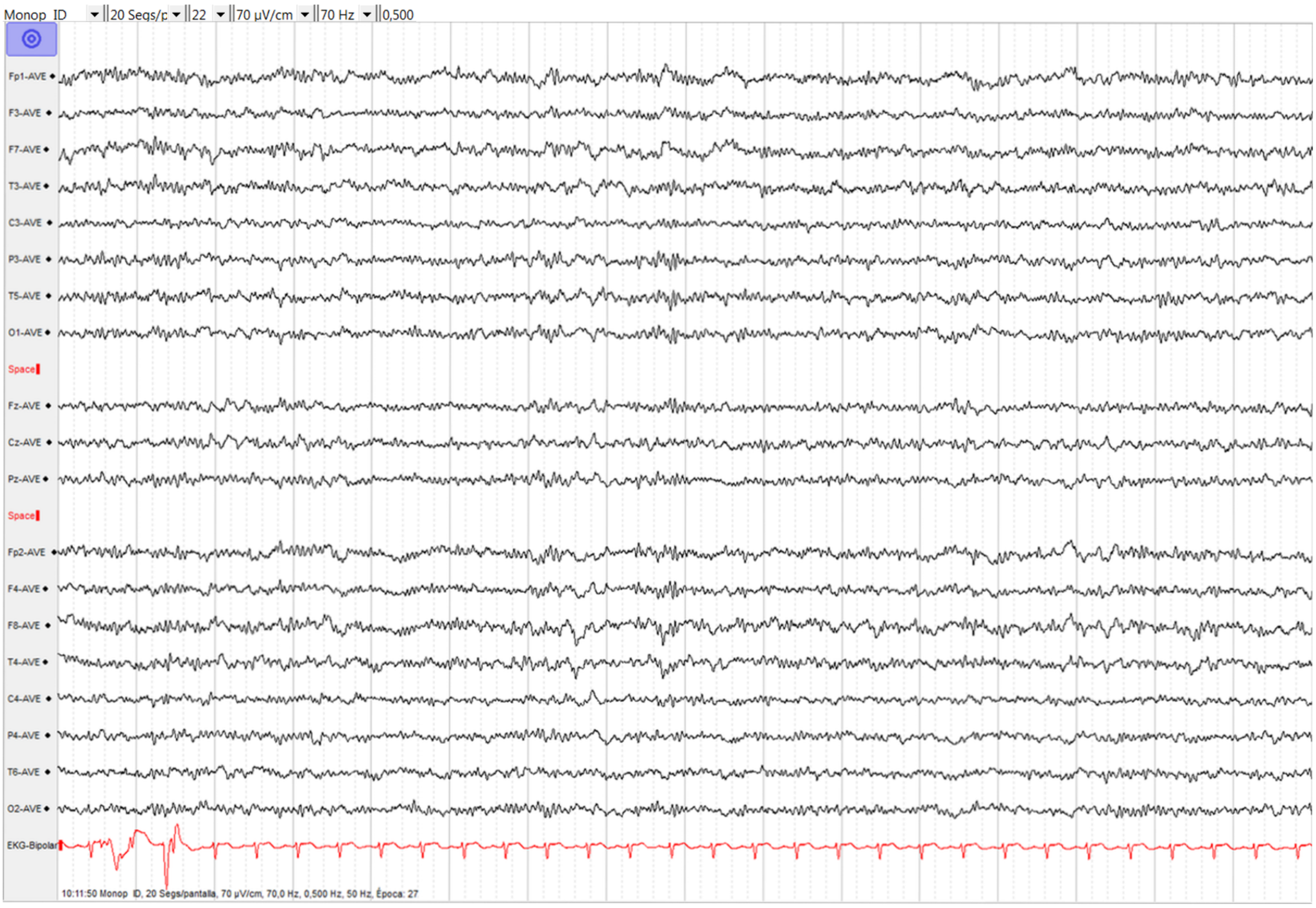

Improvement of the background activity, with difuse fast rhythms.

Patient 3

Upper image: EEG the day of the first ECT, in the ICU (before the session); lower image: EEG the day after the last ECT session.

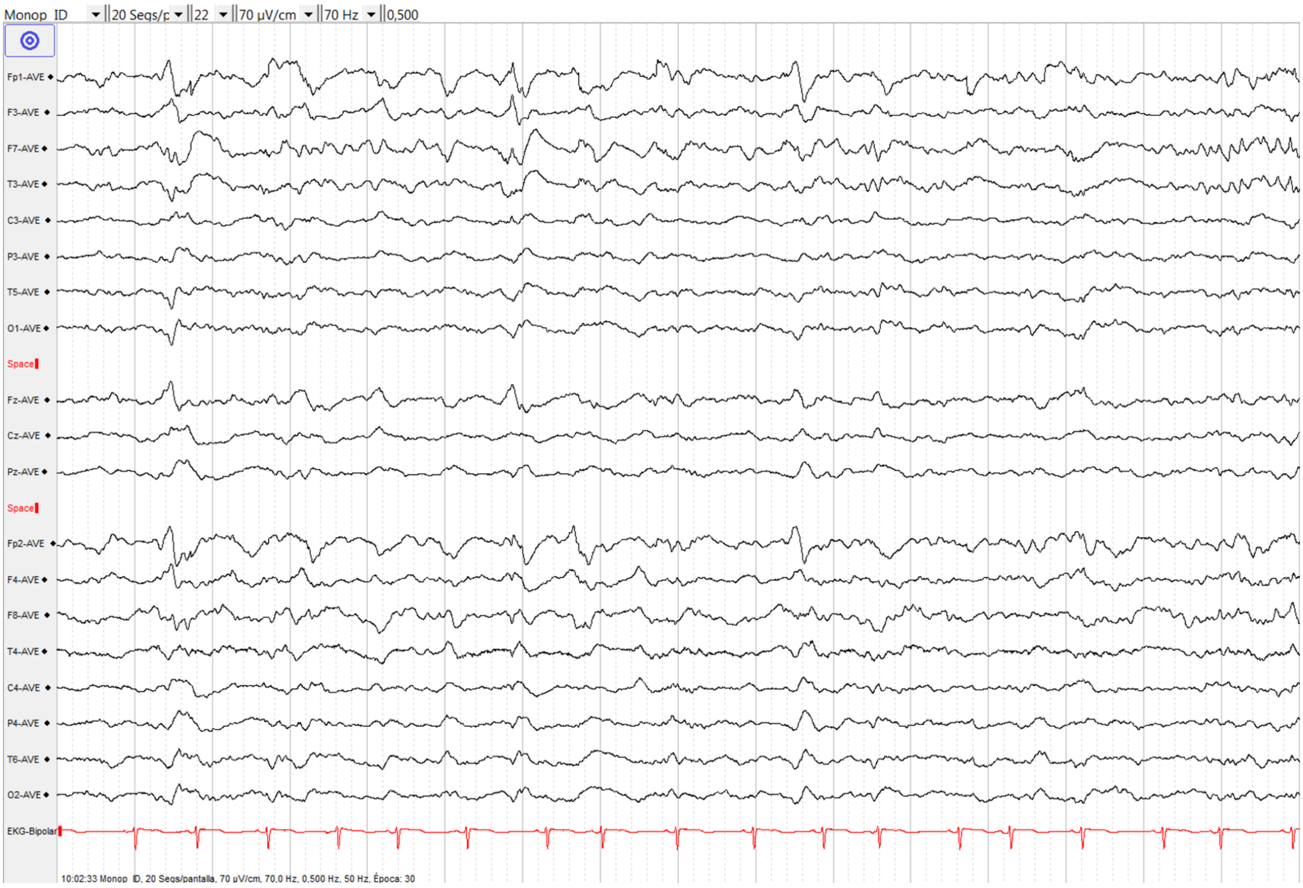

Rhytmic epileptiform discharges in frontal regions, with superimpossed sharp rhtymic frecuencies in frontal regions.

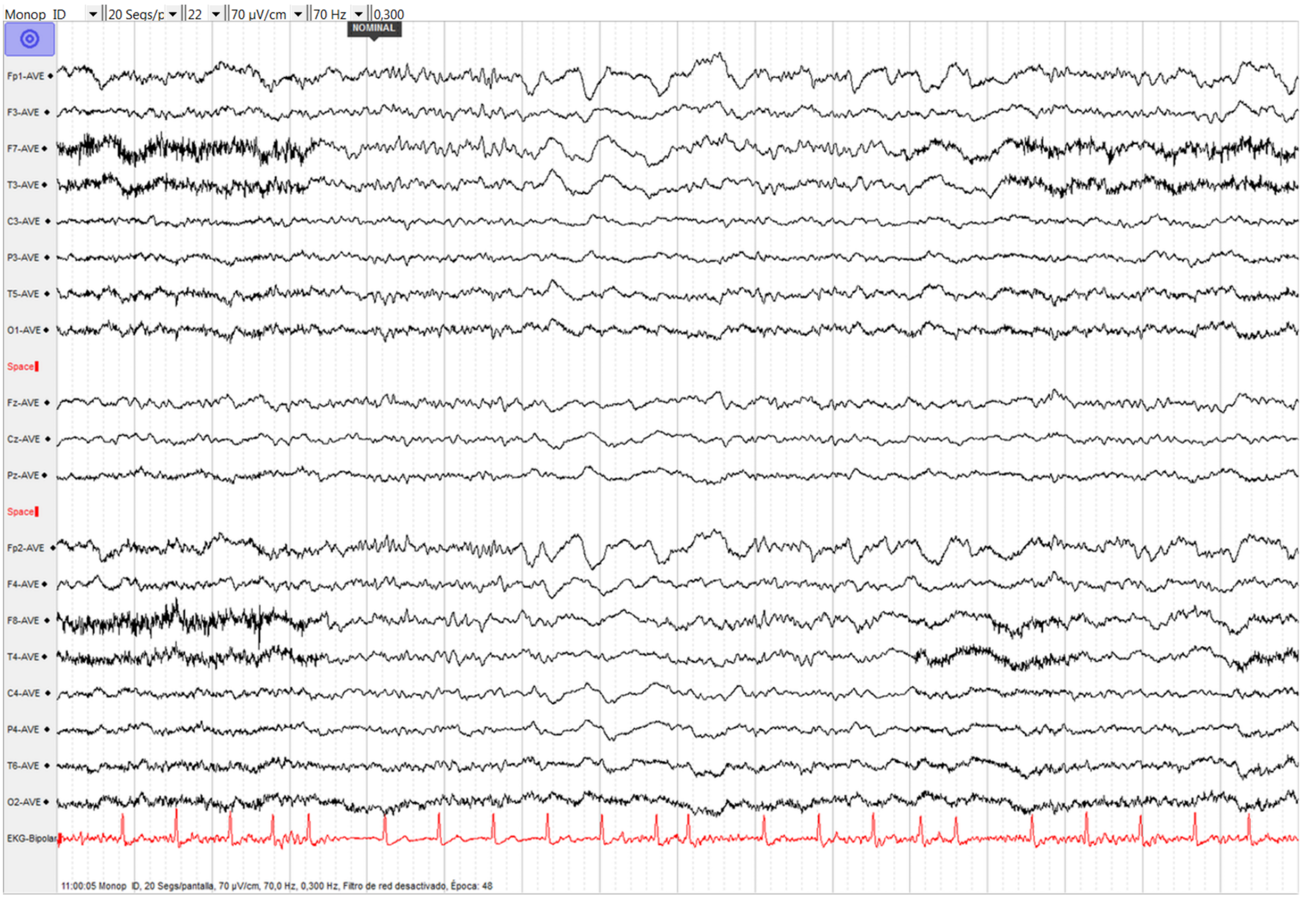

Improvement of the background activity with reactivity to nominal stimulous.

Patient 4

Upper image: EEG the day of the first ECT, in the ICU (before the session); lower image: EEG the day after the last ECT session.

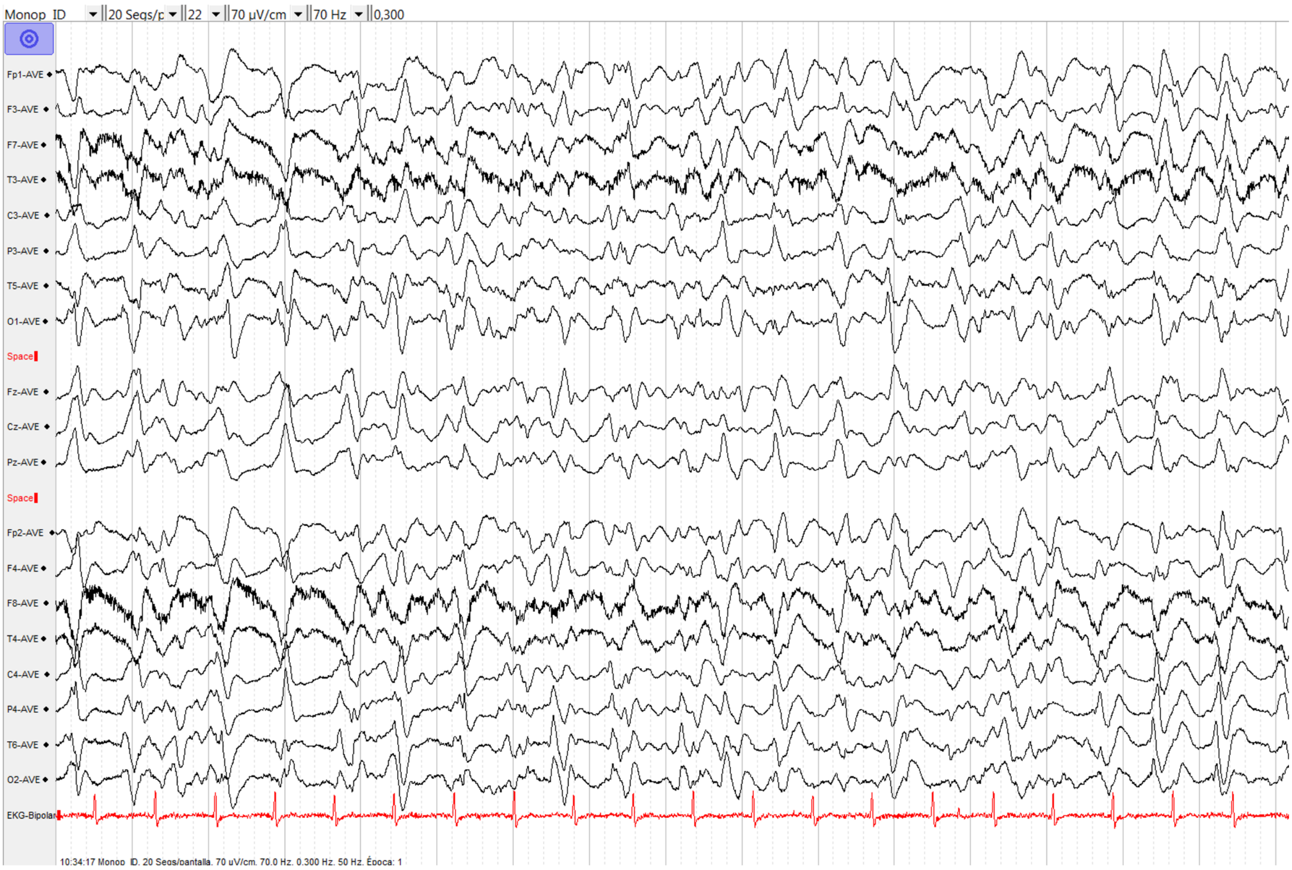

Multifocal continuos epileptiform discharges with superimpossed sharp fast rhythms.

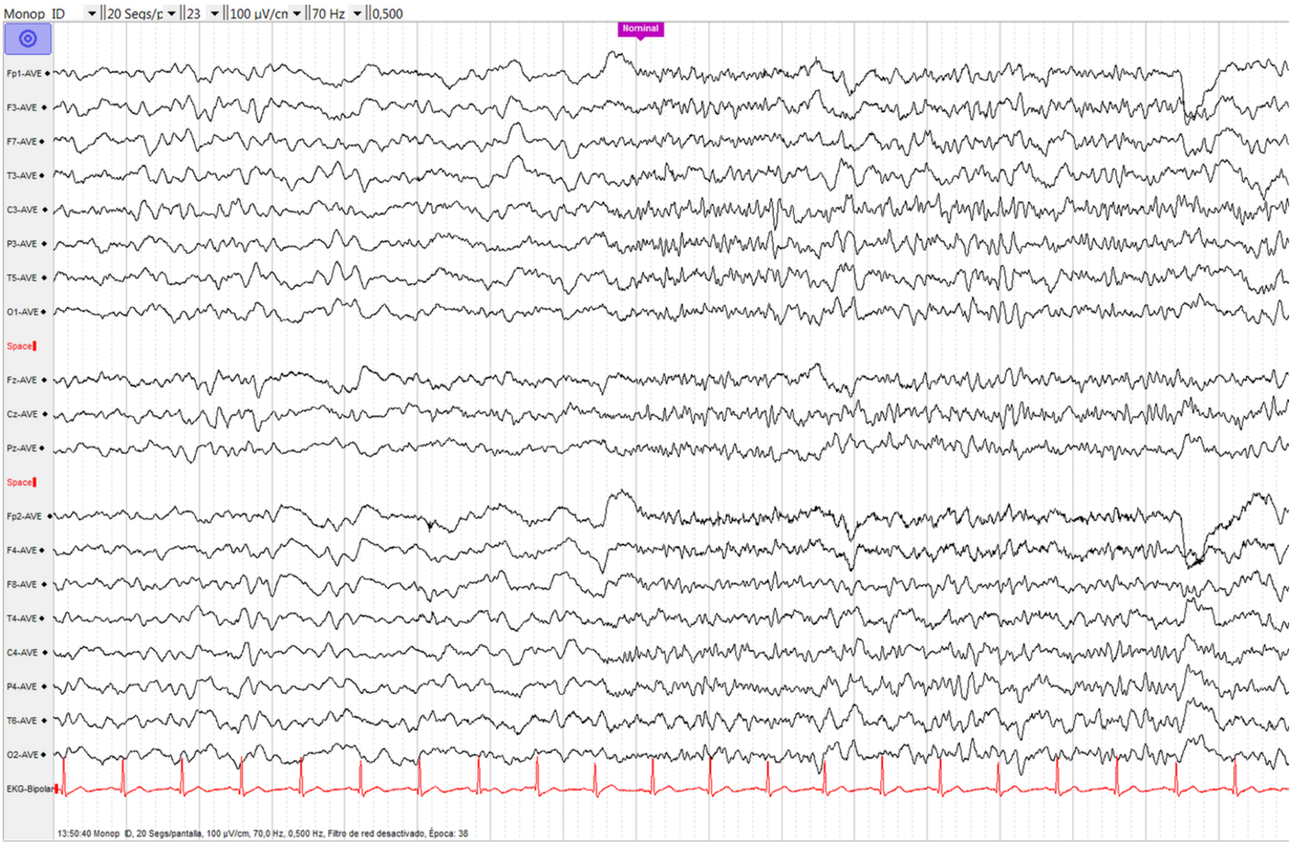

Improvement of the background activity. Reactivity to nominal stimulous.

Patient 5

Upper image: EG the day of the first ECT, in the ICU (before de session); lower image: EEG the day after the last ECT session.

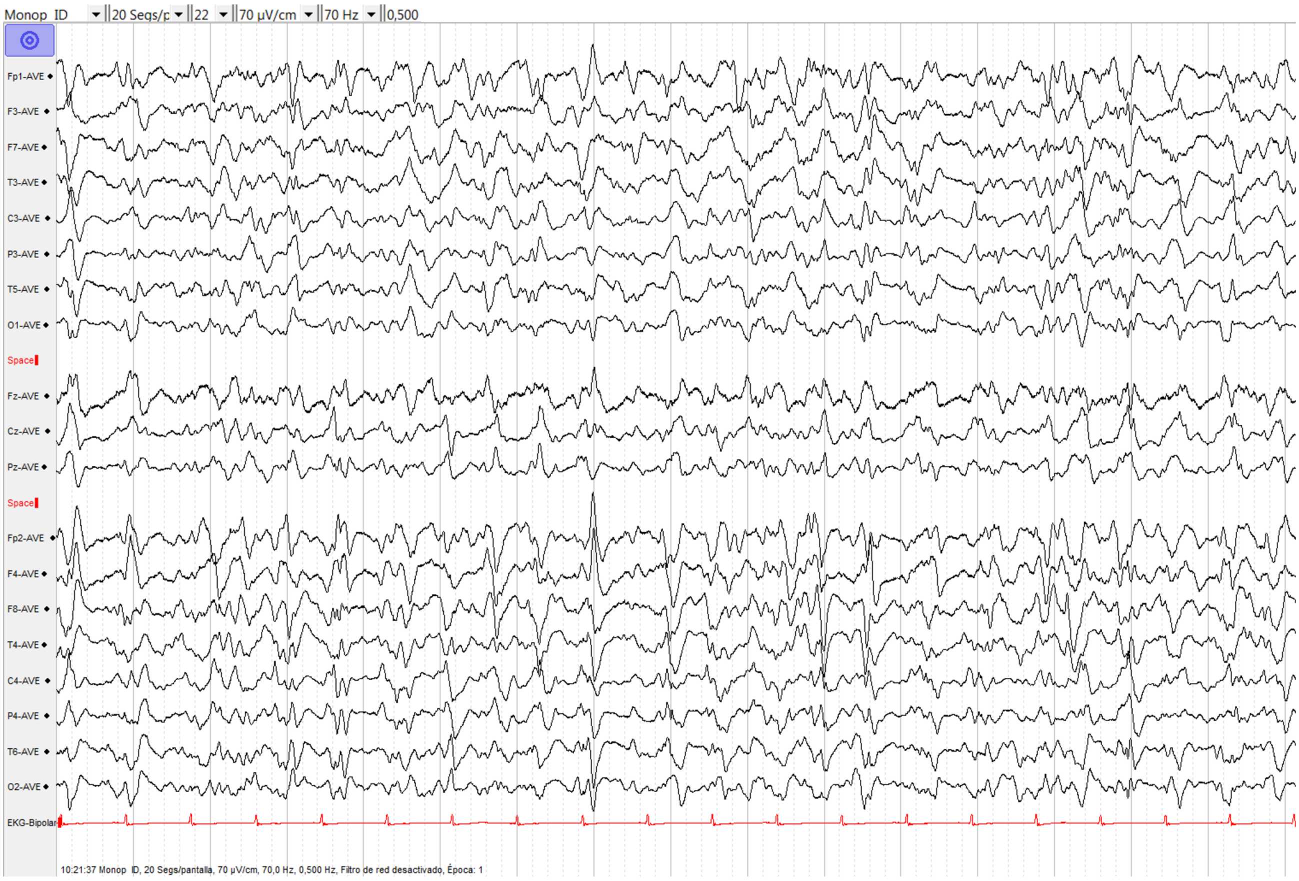

Continuous multifocal epileptiform discharges with right frontal predominance, with superimpossed sharp fast rhtyms.

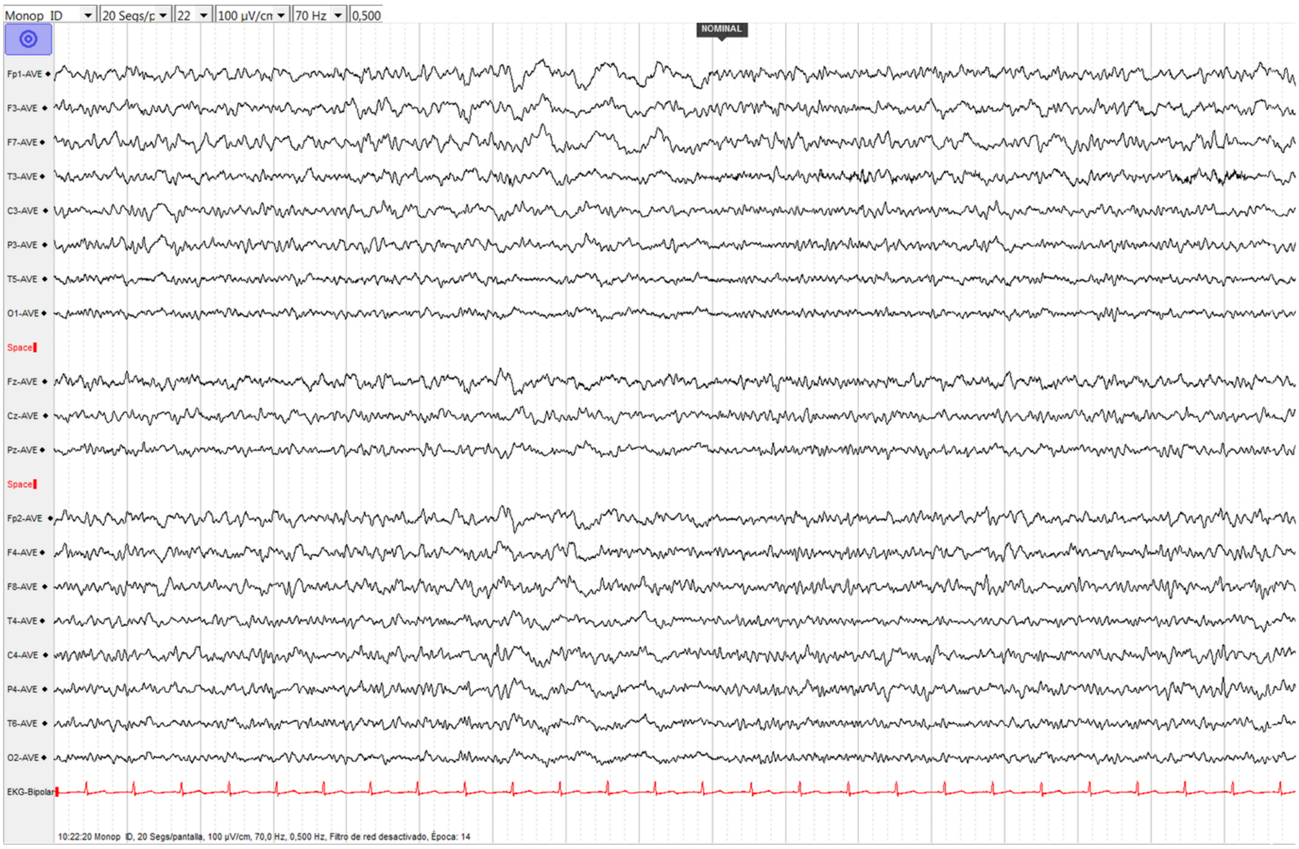

Improvement of the background activity. Reactivity to nominal stimulous.

Patient 6

Upper image: EEG the day of the first ECT, in the ICU (before de session); lower image: EEG the day after the last ECT session.

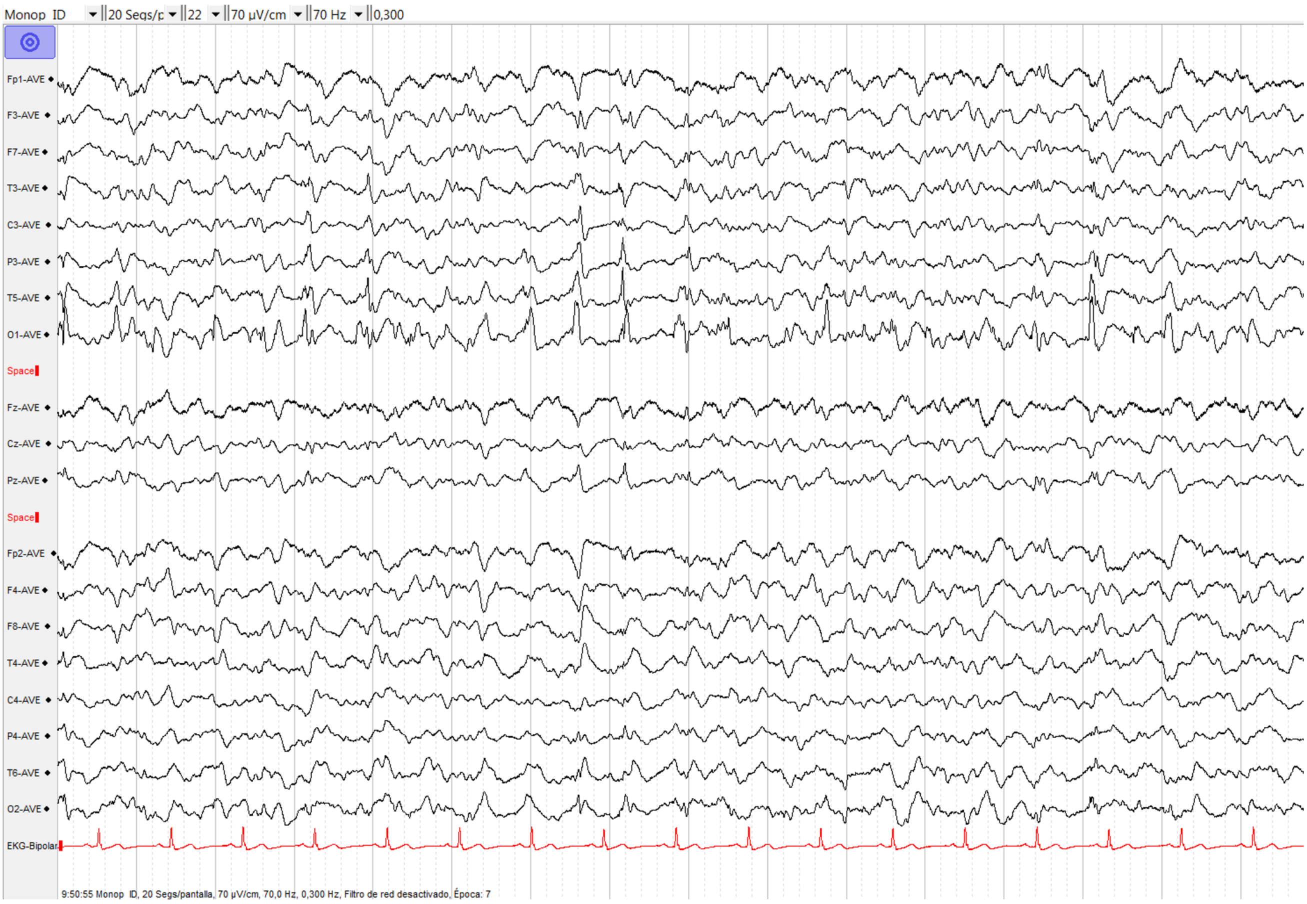

Focal continuos epileptiform discharges in left temporo-parieto-occipital regions, with superimpossed sharp fast rhythms.

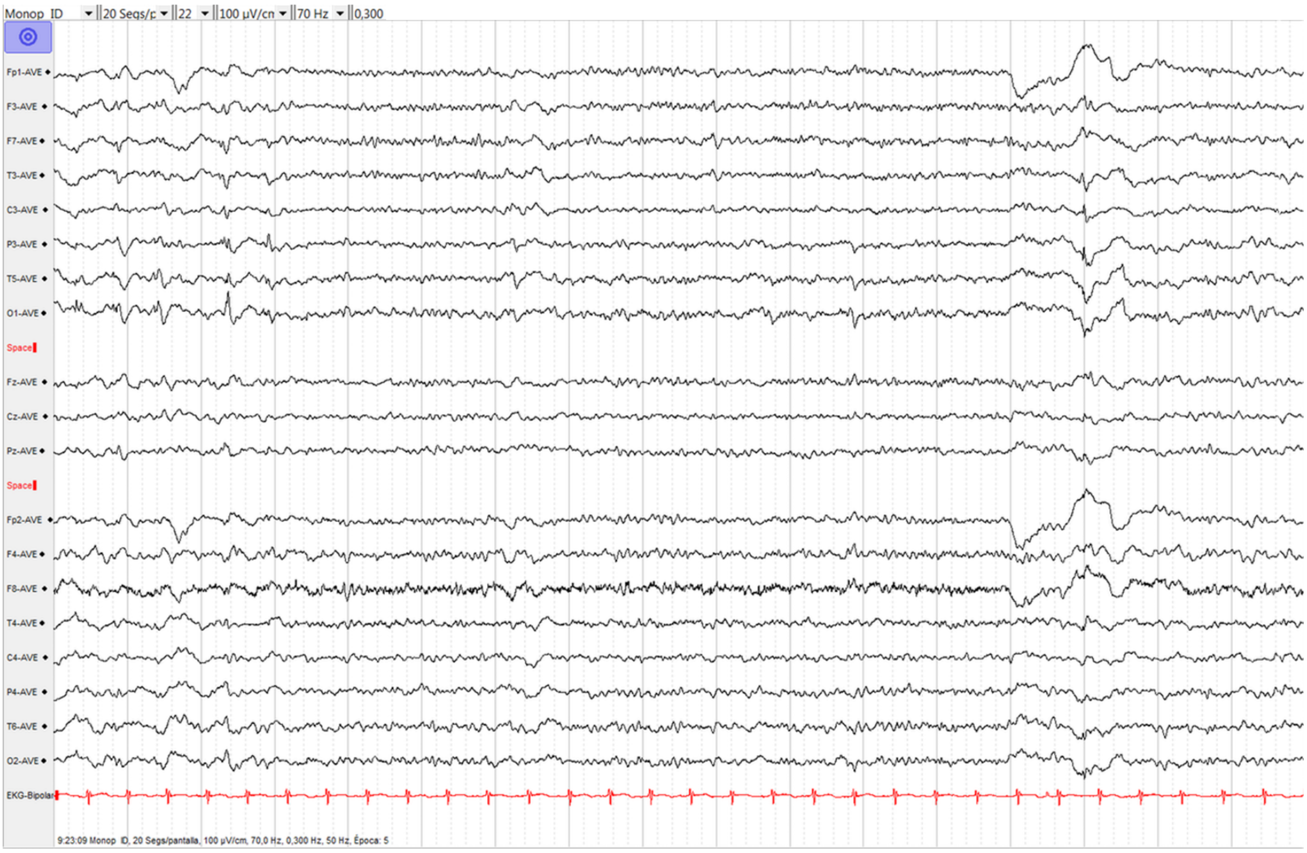

Improvement of the background activity. Interictal epileptiform discharges in left parieto-occipital regions
